# Influence of Drying Method and Argon Plasma Modification of Bacterial Nanocellulose on Keratinocyte Adhesion and Growth

**DOI:** 10.3390/nano11081916

**Published:** 2021-07-26

**Authors:** Anna Kutová, Lubica Staňková, Kristýna Vejvodová, Ondřej Kvítek, Barbora Vokatá, Dominik Fajstavr, Zdeňka Kolská, Antonín Brož, Lucie Bačáková, Václav Švorčík

**Affiliations:** 1 Department of Solid State Engineering, Institute of Chemical Technology, Prague, Technická 5, 16628 Prague, Czech Republic; vejvodok@vscht.cz (K.V.); kviteko@vscht.cz (O.K.); fajstavd@vscht.cz (D.F.); svorcikv@vscht.cz (V.Š.); 2 Department of Biomaterials and Tissue Engineering, Institute of Physiology of the Czech Academy of Sciences, Vídeňská 1038, 14220 Prague, Czech Republic; antonin.broz@fgu.cas.cz (A.B.); lucie.bacakova@fgu.cas.cz (L.B.); 3 Department of Biochemistry and Microbiology, Institute of Chemical Technology, Prague, Technická 5, 16628 Prague, Czech Republic; vokataa@vscht.cz; 4 Materials Centre of Usti nad Labem, Faculty of Science, J. E. Purkyně University, Pasteurova 15, 40096 Ústí nad Labem, Czech Republic; zdenka.kolska@ujep.cz

**Keywords:** bacterial nanocellulose, lyophilization, plasma modification, cell adhesion

## Abstract

Due to its nanostructure, bacterial nanocellulose (BC) has several advantages over plant cellulose, but it exhibits weak cell adhesion. To overcome this drawback, we studied the drying method of BC and subsequent argon plasma modification (PM). BC hydrogels were prepared using the *Komagataeibacter sucrofermentans* (ATCC 700178) bacteria strain. The hydrogels were transformed into solid samples via air-drying (BC-AD) or lyophilization (BC-L). The sample surfaces were then modified by argon plasma. SEM revealed that compared to BC-AD, the BC-L samples maintained their nanostructure and had higher porosity. After PM, the contact angle decreased while the porosity increased. XPS showed that the O/C ratio was higher after PM. The cell culture experiments revealed that the initial adhesion of human keratinocytes (HaCaT) was supported better on BC-L, while the subsequent growth of these cells and final cell population density were higher on BC-AD. The PM improved the final colonization of both BC-L and BC-AD with HaCaT, leading to formation of continuous cell layers. Our work indicates that the surface modification of BC renders this material highly promising for skin tissue engineering and wound healing.

## 1. Introduction

Bacterial (or microbial) nanocellulose (BC) has been known for more than two thousand years as a by-product of the kombucha tea fermentation process [1], although the first person to scientifically observe and describe this material was A. J. Brown in 1886. During his work with *Bacterium acetum*, he described it as a translucent jelly-like material that occurs on the surface of the cultivation fluid and proved it to be cellulose [2]. Nowadays, several gram-negative aerobic rod-like bacteria genera with high acid tolerance producing BC are known, especially the most efficient producer of BC—*Komagataeibacter* (formally known as *Gluconacetobacter* or *Acetobacter*) [3,4,5]. On the liquid surface, the bacteria form BC that protects them from dry-out, irradiation, lack of oxygen, and pathogens [6]. Many examples of scientific articles describing the production of BC using various bacterial species and subspecies, media composition (carbon and nitrogen source, pH), and reaction conditions leading to materials of various shapes and properties can be found in literature [7,8,9].

Although BC has the same chemical composition as plant cellulose (PC), it differs significantly in its other properties. BC is obtained in higher purity since there is no need for the removal of other plant polymers [10]. It consists of fibres that are thinner than 100 nm (compared to PC having fibres the size of around 30 µm [11]) so it can be classified as nanocellulose. This nanostructure provides BC with its remarkable properties such as high porosity, specific surface area, crystallinity (60–80% [12,13]), water-holding capacity, permeability for gases and liquids, and excellent mechanical properties [14,15]. The degree of polymerization of BC is usually between 2000 and 6000 [16].

Bacterial nanocellulose and its composites have already been used in many fields of industry such as fashion [17], papermaking and packaging [18,19], audio membranes [20], air purification membranes [21], and cosmetics [22]. BC has also been used in medicinal applications [23] such as drug delivery vehicles (e.g., propranolol [24], ibuprofen, lidocaine [25], doxorubicin [26]), ophthalmology [27,28], and regenerative medicine [29,30,31,32]. Nowadays, there are several BC-based commercially available wound dressings [33] that are supposed to treat ulcers, burns, or chronic wounds such as Bionext^®^ [34], XCell^®^ [35], Nexfill^®^, Nanoderm^TM^ [36] and Dermafill^®^ [37]. These materials induce epithelialization with no need for everyday re-dressing [23] thanks to their permeability for air and liquids [12,38]. BC has gained popularity in these medicinal applications thanks to its non-pyrogenicity, non-toxicity, biocompatibility, similarity to soft skin tissue, and ability to provide an optimal three-dimensional substrate for cell attachment [12,23,39].

Although BC exhibits good biocompatibility [40] leading to the above mentioned medicinal applications, it exhibits quite poor cell adhesion, which can be improved by several different methods: (1) immobilization of various adhesion proteins, (2) preparation of BC-based composites with various biomolecules such as gelatin or collagen, (3) by plasma surface modification, (4) or by tailoring the surface properties such as porosity or morphology [41].

Plasma modification (PM) is a method that can be used to modify the biomaterial surface into the depth of about 1 nm while maintaining the properties of the bulk material. This leads to a biomaterial with altered surface properties such as morphology, chemical composition, and hydrophilicity but with preserved mechanical properties and functionality [42,43]. Alteration of these surface properties can lead to enhanced biocompatibility and cell adhesion, since the modified surfaces provide a better cell support, e.g., by improved absorption of cell adhesion-mediating proteins [10]. BC has been so far modified with nitrogen [44], oxygen and fluoromethane [45] plasmas. The cited studies observed that plasma-modified BC contains higher concentration of functional groups improving the cell adhesion, has higher porosity and lower water contact angle. These changes resulted in improved cell adhesion.

For PM and manipulation of the surface properties generally, it is important that the sample is properly dried. After harvesting and washing the BC hydrogel, the excessive water can be removed by several drying methods: air drying, oven drying, draining with water-absorbing material, supercritical drying, or lyophilization. Generally, lyophilization and supercritical drying are milder drying methods; the material maintains its nanostructure, and therefore shows a higher water swelling ratio and porosity [6,8,9,46,47].

**Figure 1:**
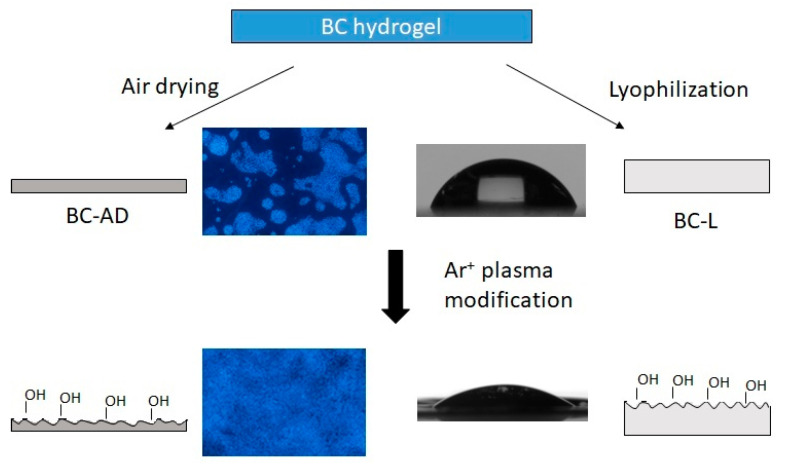
Diagram of the mechanism for improving the cell adhesion of bacterial nanocellulose.

In this work the influence of different drying methods of BC with subsequent PM on the surface properties, morphology, and keratinocyte adhesion was studied (for the diagram of the work see Figure 1). Since the cell adhesion on native BC is quite poor, we chose the Ar^+^ PM as a cheap, quick, proven, and easy method to control the surface properties. The plasma-modified samples exhibited a higher porosity due to the etching and a lower contact angle. This method has been shown to be able to enhance the cell adhesion on BC.

## 2. Materials and Methods

### 2.1. Preparation of BC Foils

Bacterial nanocellulose was produced by *Komagataeibacter sucrofermentans* (Leibniz-Institut DSMZ, Braunschweig, Germany, DSM 15973). Cultivation was carried out in Hestrin-Shramm [48] culture medium, consisting of D-glucose (20 g/L), disodium hydrogen phosphate dodecahydrate (6.8 g/L), special peptone (5 g/L), yeast extract (5 g/L), and citric acid monohydrate (1.3 g/L), pH 5.8. Cultivation lasted for at least 7 days at 28 °C in Erlenmeyer flask statically. Purification of the nanocellulose from the bacteria and medium residue was performed by rinsing in boiling 0.1 M NaOH two times and then rinsing in boiling distilled water two times. To fully remove the bacteria residue, we washed the samples in 10% (m/m) solution of SDS and trypsin solution. Washed BC hydrogels were solidified via air-drying on PTFE foil (AD) or lyophilization (L) (FreeZone 2.5, Labconco, Kansas City, MO, USA) for at least 24 h. Circular samples with a diameter of 16 mm were then cut from the dried BC foils.

### 2.2. Plasma Modification of BC Foils

The surface of the solid samples was modified in a direct (glow, diode) Ar^+^ plasma discharge on Balzers SCD 050 device (BAL-TEC, Balzers, Lichtenstein). The conditions were set as follows: gas purity 99.997%, pressure of 7 Pa, electrode distance of 55 mm, electrode area of 48 cm^2^, chamber volume of approx. 1 dm^3^, plasma volume of 0.24 dm^3^, electrical current of 15 mA, and voltage of 680 V. The samples were modified from both sides for several different exposure times (60 s, 240 s, and 480 s). The samples were then named BC-AD 60 s, BC-AD 240 s, and BC-AD 480 s and BC-L 60 s, BC-L 240 s, and BC-L 480 s for BC-AD and BC-L, respectively.

### 2.3. Methods of Analysis

The thickness of the dried BC foils was measured with digital caliper micrometer QuantuMike IP65 (0–25 mm, 0.001 mm, Mitutoyo, Kawasaki, Japan). The measurement was repeated for at least 20 different areas on each sample.

Chemical composition of materials and evaluation of chemical changes after freeze-drying and plasma modification were measured by Fourier transform infrared spectroscopy (FTIR—ThermoFisher, Nicolet iS5 with iD7 attenuated total reflection accessory with diamond crystal, Waltham, MA, USA). The spectra were obtained as an average from 128 measurement cycles with a spectral range of 600–4000 cm^−1^, and 1 cm^−1^ data interval. The changes of surface composition were also studied by X-ray photoelectron spectroscopy, ESCAProbe P (Omicron Nanotechnology, GmbH, Taunusstein, Germany) device was used for these measurements using a monochromatic energy source at 1486.7 eV. The exposed and analyzed area was 2 × 3 mm^2^ and the spectra were obtained with 0.05 eV energy step. CasaXPS software was used to analyze the spectra.

The surface morphology of BC nanofibers was studied using a dual-beam focused ion beam-scanning electron microscope with a FEG electron gun (FIB-SEM TESCAN LYRA3GMU, Brno, Czech Republic). The sample morphology was investigated using SEM at an acceleration voltage of 7 keV and a deceleration voltage of 5 keV. Prior to the measurement, the samples were sputtered with a thin film of platinum and attached to the sample holder with silver paint to avoid surface charging during the measurements.

Water contact angles of BC samples were measured by the sessile drop method in order to evaluate the changes in surface hydrophilicity by fully automated goniometer DSA100 (KRÜSS GmbH, Hamburg, Germany). A 2 µL droplet of distilled water was produced on a capillary tip above the studied surface of BC and after 5 s it was carried to the surface. The whole sequence was recorded on video at a recording speed of 50 fps. The contact angle was calculated from the captured frame where the sessile drop just spread over the surface to its highest diameter to eliminate the influence of the water being absorbed into the material.

Surface area and pore volume were determined by N_2_ adsorption/desorption isotherms. Samples were degassed at room temperature for 24 h. After that, adsorption and desorption isotherms were measured with nitrogen (N_2_, Linde, 99.999% purity) using Quantachrome Instruments NOVA3200 (Anton Paar GmbH, Graz, Austria). All samples were measured three times with an experimental error of less than 5%. Five-point Brunauer-Emmett-Teller (BET) analysis has been applied to determine the total surface area and a 40-points Barrett-Joyner-Halenda (BJH) model was used for determining the pore volume. 

Gravimetric analysis was used to get the material loss after plasma modification. Samples were weighed before and after plasma modification at least five times each on the UMX2 ultra-microbalance system (Mettler Toledo, Greifenses, Switzerland). The weight loss Δm was calculated using this equation: Δm = (m_0_ − m_1_)/m_0_, where m_0_ and m_1_ stand for weight before and after modification, respectively.

### 2.4. Cell Model and Culture Conditions

The human keratinocytes of the line HaCaT, purchased from CLS Cell Lines Service (Eppelheim, Germany), were cultivated in Dulbecco’s Modified Eagle’s Medium (DMEM; Sigma-Aldrich Co., St Louis, MO, USA) with 10% of fetal bovine serum (FBS; Sebak GmbH, Aidenbach, Germany) and 40 μg/mL of gentamicin (Novartis International AG, Basel, Switzerland).

Selected circular BC samples (BC-AD and BC-L, either unmodified or modified with plasma for 240 s) were sterilized with in an autoclave (121 °C, 23 min, 101.3 kPa) and inserted into the wells of 24-well cell culture polystyrene plates (TPP, Trasadingen, Switzerland). The cells were seeded on the samples at a density of approximately 15,000 cells/cm^2^ (i.e., 30,000 cells/well) into 1.5 mL of the cell culture medium (mentioned above) per well. The cells were then cultivated for three time periods (1, 4, and 7 days) at 37 °C and in a humidified air atmosphere with 5% CO_2_. Tissue culture polystyrene (PS) wells were used as reference material.

### 2.5. Evaluation of the Cell Number, Morphology, and Viability

The number and morphology of HaCaT cells on BC samples and control PS wells were evaluated on days 1, 3, and 7 after seeding. First, the cells were rinsed with phosphate-buffered saline (PBS) and were fixed with −20 °C cold ethanol for 5 min. Then, the cells were incubated with a combination of fluorescent dyes diluted in PBS, namely Hoechst 33258, which stains the cell nuclei (5 μg/mL; Sigma-Aldrich, Schnelldorf, Germany), and Texas Red C_2_-maleimide, which stains the proteins of the cell membrane and cytoplasm (20 ng/mL; Thermo Fisher Scientific, Waltham, MA, USA), for 1 h at room temperature in the dark. Images of the cells were taken using an epifluorescence microscope (IX 51; Olympus, Tokyo, Japan; objective 4×), equipped with a digital camera (DP 70). On day 1 after seeding, the number of human HaCaT keratinocytes was evaluated by direct counting on the images taken under the fluorescence microscope using the ImageJ software. In the following days (days 4 and 7), when the direct cell counting was disabled by the increasing cell density and cell overlapping, the cell number was estimated indirectly by measuring the intensity of fluorescence of Hoechst 33258-stained cells on microphotographs, taken at the same exposure time for all experimental groups, using ImageJ software.

The viability of HaCaT cells was measured on day 4 after seeding by a trypan blue-exclusion test in an automated Vi-Cell XR Cell Viability Analyser (Beckman Coulter, Indianapolis, IN, USA) from four parallel samples of each experimental group. Before the analysis, the cells were detached from the material by incubation in a trypsin-EDTA solution (0.05% trypsin, 0.02% EDTA, Sigma-Adrich, Schnelldorf, Germany) for 8 min at 37 °C in a humidified air atmosphere with 5% CO_2_.

### 2.6. Statistics

Quantitative data are presented as arithmetic mean ± standard deviation values (S.D.) or standard error of the mean (S.E.M.) from three or more independent samples for each experimental group. Statistical significance was evaluated using SigmaPlot 14.0, analysis of variance, Student–Newman–Keuls method, or nonparametric Kruskal–Wallis test. Values of *p* ≤ 0.05 were considered significant.

## 3. Results and Discussion

### 3.1. Production of BC Pellicles

**Figure 2:**
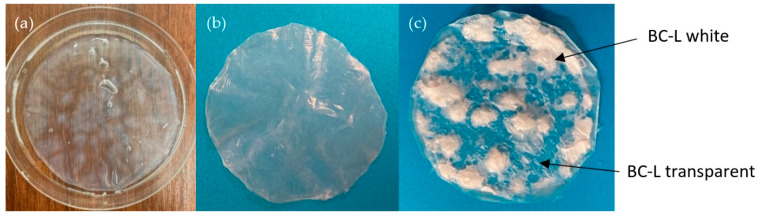
(**a**) Harvested and washed bacterial nanocellulose (BC) pellicle; (**b**) air-dried BC pellicle; (**c**) lyophilized BC pellicle.

Bacteria of the *Komagataeibacter sucrofermentans* (ATCC 700178, DSM 15973) strain were cultivated for at least 7 days to obtain BC pellicles. Literature sources suggest 7-day cultivation to be sufficient for BC production due to the declining carbon source after this time [49,50,51,52]. After harvesting and washing as reported above, we obtained transparent hydrogel of total mass approx. 23 mg out of 100 mL of medium. The yield did not increase further with time. This hydrogel had an uneven surface (Figure 2a). We suggest this to be caused by the non-homogeneous distribution of bacteria cells in the pellicle during cultivation, which leads to thicker (cloudy) and thinner (transparent) parts. These heterogeneities are also visible on the lyophilized BC (BC-L) (Figure 2c), where the cloudy parts are changed into white opaque material, while the transparent parts remain as they were. The air drying (AD) was carried out on a hydrophobic PTFE foil to prevent the material from sticking to the drying pad (Figure 2b). This BC-AD did not show any visible heterogeneities in the surface. These two drying methods also showed differences between the thickness of the material, which was (10.55 ± 3.99) µm for BC-AD, (19.45 ± 6.41) µm for the transparent part of BC-L, and (51.45 ± 13.53) µm for the white parts of BC-L. These results follow the trend suggested by Illa [47] and Vasconcellos [46] that the freeze-drying preserves the morphology of the original hydrogel and therefore the film thickness is significantly higher compared to AD (resp. oven dried in their case). Zeng [8] did not observe such significant differences between AD and L. This could be caused by selection of different bacterial strain which resulted in different morphology of the material. Compared to Illa [47], we did not observe differences between the fiber diameters. Our material showed fiber diameter of (58.22 ± 14.47) nm for BC-AD, (65.21 ± 15.00) nm for the transparent part of BC-L, and (54.88 ± 14.57) nm for the white part of BC-L. These results are in accordance with those observed by Zeng [8]. For the following experiments circular samples with diameter of 16 mm were cut. There was roughly the same amount of white and transparent parts in these BC-L circles.

### 3.2. Surface Modification of BC

In this work we modified both BC-AD and BC-L samples with Ar^+^ plasma with the power of 7 W. We used three different exposure times to evaluate the effects of the modification process. Samples were modified from both sides for easier further manipulation. The differences were evaluated by gravimetric analysis, FTIR, and XPS to examine the composition changes, SEM for morphology changes, and contact angle to determine the hydrophilicity.

**Figure 3:**
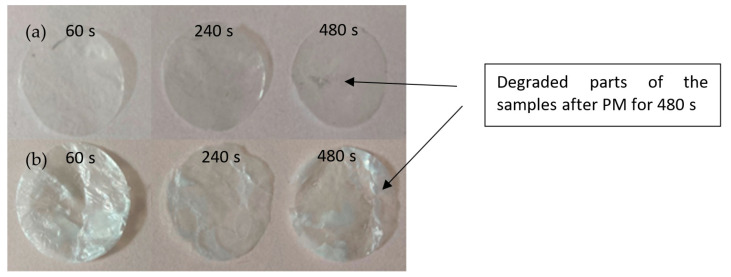
Samples after plasmatic modification for various exposure times (60–480 s): (**a**) BC-AD, (**b**) BC-L. The samples with 480 s PM shows signs of degradation.

The Ar^+^ PM leads to surface ablation of the samples (plasma etching). The rate of the ablation depends on the chemical composition of the polymer chain and properties of the used plasma discharge (plasma type, composition, discharge power, and exposure time) [53]. Almost all the modified samples showed degradation to various extents. Sample edges were burned and turned brown-blackish, but the central parts of the samples remained intact. This was, however, not the case of the samples modified for 480 s that showed signs of degradation over the whole area of the samples. The middle part degraded with much higher intensity, burned holes in the samples were observed (Figure 3). Gravimetric analysis (Table 1) showed that these samples have significantly higher weight loss. Therefore the samples with 480 plasma exposure time were not further examined, since the degradation leads to very inhomogeneous sample surface. Overall, the BC-AD samples showed higher rate of weight loss. This could be caused by the removal of larger parts of the material during PM due to their more compact and at the same time brittle nature.

### 3.3. Chemical Composition of BC

**Figure 4:**
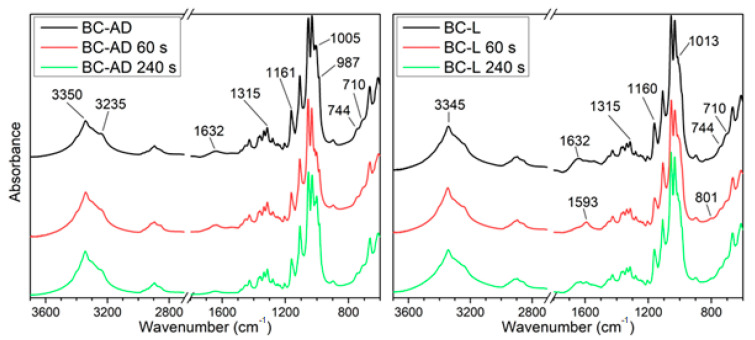
FTIR absorbance spectra of the BC-AD and BC-L unmodified and plasma-modified samples.

FTIR spectra of the BC-AD and BC-L samples unmodified and after 60 s and 240 s PM are shown in Figure 4. Spectral interpretation and band assignment in cellulose is considered to be somewhat problematic due to the dominant role of inter- and intra-chain hydrogen bond interactions that lead to numerous combination vibrations [54]. However, some general features of the material can be ascribed to the absorption bands in the present spectra. The spectra of the prepared BC correspond to cellulose I structure with both Iα and Iβ components being present, which is confirmed by the presence of weak absorption bands at both 744 and 710 cm^−1^. This can be assigned to the glucose ring deformations, compounded with glycosidic bond bending of the respective cellulose structure modifications [54].

The OH band around 3350 cm^−1^ combines the vibrations of the hydrogen bonds in cellulose. The absorption at 3235 cm^−1^, which is slightly stronger in the case of the BC-AD sample is usually attributed to the 2O-H···6O-H···3O hydrogen bond group [55].

The absorption in the 1700–1500 cm^−1^ range relates to adsorbed water [56]. While all the BC-AD samples show a single weak absorption at 1632 cm^−1^, in the BC-L samples the absorption is noticeably stronger and the absorbed range is wider, in certain cases with a dominant second absorption maximum at 1593 cm^−1^. This indicates there is a higher amount of adsorbed water in the BC-L samples and the water binds to the cellulose structure differently than in the air-dried samples.

The absorption band at 1315 cm^−1^, which can be assigned to C-O-H bending vibrations [57], is relatively stronger in the BC-AD samples. The only absorption band where a consistent shift with the PM can be observed was the 1160 cm^−1^ which shifts about 2 cm^−1^ to lower wavenumber in samples after PM. Moreover, this band appeared on average about 20% weaker in those samples. These changes are in the scale of the whole spectrum rather insignificant; however, this can be expected, since PM mainly influences the very surface layer of the material and the FTIR signal is obtained over the sample depth of several micrometers. Nonetheless, the variation of this absorption band could mean the glycosidic bond and the cellulose chains have been to some degree disrupted by the PM. The air-dried samples also show weaker maxima at 1005 and 987 cm^−1^ (bands related to vibrations of C-O bonds in the hydroxyl groups), while in the lyophilized samples these absorptions show merely as shoulders of the stronger bands. Especially in the case of the air-dried samples, the absorption at 1005 cm^−1^ shows very high variability, even within repeated measurements on a single sample. In the case of the BC-L samples with PM, a weak band was observed around 800 cm^−1^. Absorptions in this region are usually assigned to deformations of the glucose ring coupled with bending of the glycosidic bond. Therefore, this could indicate the influence of PM on the glycosidic bond in the BC samples.

XPS measurements showed the changes in the surface composition, especially in the carbon and oxygen content. The samples were somewhat contaminated with Al, Si, F, S, Na, and N from the plasma vacuum chamber, and the bacteria and medium residues. The results shown in Table 2 are recalculated without the contaminating elements. The expected value for O/C from the chemical structure of cellulose is 0.83. For unmodified samples, this value is slightly lower, which is caused by the residues of media and bacteria on the surface of the material. The most significant result is the large increase in oxygen content after PM of BC-L 240 s. This could be caused by the disruption of the cellulose structure leading to the formation of highly reactive species on the surface, which react with ambient oxygen after the exposure of the modified surface to atmosphere. The increase in oxygen content after PM for BC-AD is also apparent.

### 3.4. Surface Morphology of BC

**Figure 5:**
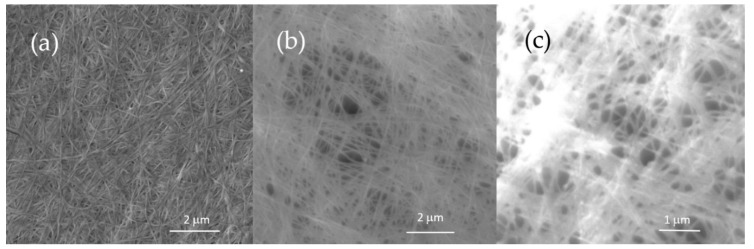
Nanostructure images of BC samples analyzed by SEM: (**a**) BC-AD, (**b**) BC-L transparent, (**c**) BC-L white.

The SEM images in Figure 5 show the nanostructured morphology of unmodified BC-AD and BC-L (transparent and white part) samples. The differences between BC-AD and BC-L samples can be seen in the porosity of the material. For the L samples, the fibers are more spread out. This corresponds with the fact that the lyophilized pellicles are thicker. Illa [47], who compared lyophilization and oven drying, suggested that this phenomenon is caused by the free hydroxyl groups that can form secondary bonds. The mobility of the amorphous regions during freezing is reduced and therefore the morphology is preserved. However, during oven drying the thermal energy maintains the mobility of the amorphous chains and therefore the morphology collapses, the fibers come closer together, and those samples are thinner. Air drying at room temperature could have similar effect on the material morphology. Together with the smaller thickness of the material compared to BC-L, SEM shows that BC-AD has a more rugged surface structure with the bent fibers turned perpendicular to its surface. The differences between SEM images of BC-L transparent and BC-L white were studied as well. Those nanofibers were found to be indistinguishable in the SEM images for both samples. The plasma-modified samples did not show any differences in these areas either, so we can suppose that the thickness of the lyophilized pellicles does not affect the nanofibrillar structure and it is determined by the bacterial origin of the material.

**Figure 6:**
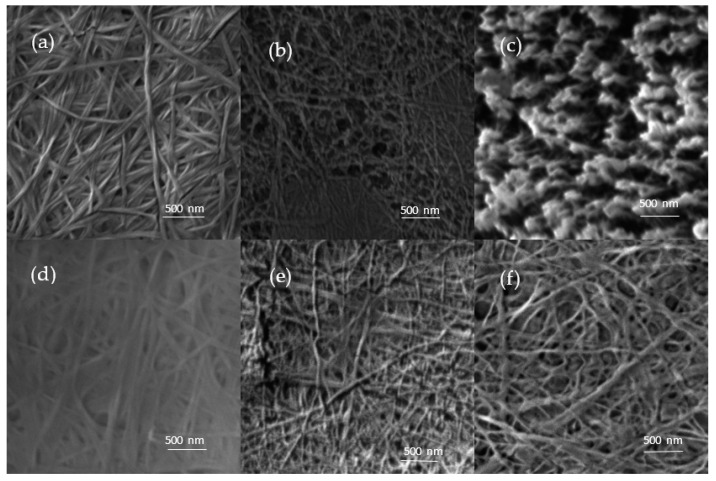
BC before and after plasmatic modification as analyzed by SEM: (**a**) BC-AD, (**b**) BC-AD 60 s, (**c**) BC-AD 240 s, (**d**) BC-L, (**e**) BC-L 60 s, (**f**) BC-L 240 s.

It has been demonstrated that PM can change the material surface morphology, especially the surface roughness and porosity. This can lead to a material with different surface properties such as hydrophilicity [58]. The SEM analysis revealed ablation occurring, which is caused by surface etching during PM (Figure 6). The cellulose fibers appeared to be rugged and thinner after PM. Observed surface-terminated pores were bigger in the case of BC-AD. Those pores were wider and deeper with higher PM exposure time. The pores of BC-AD 240 s merged, forming a “brush” from the remaining parts of the fibers on surface. This very different structure is in agreement with the fact that the BC-AD 240 s sample was modified to the highest degree of the compared samples based on the gravimetric analysis. This is caused by the higher exposure time (compared to BC-AD 60 s) and by the fact that fibres in the air-dried sample are closer to each other than in the lyophilized ones. These results are in agreement with those published by Pertile et al. [44].

### 3.5. Gas Sorption Analysis of Porosity and Specific Surface Area

Specific surface area of unmodified samples and samples with 240 s PM were measured by gas sorption method. These results (Table 3) are in good agreement with the SEM observations. Firstly, compared to BC-AD, BC-L has almost five times higher porosity and specific surface area, because it maintains its structure (pellicle thickness) during the drying process. Further increase of pore volume and surface area was obtained after PM. This is due to PM fraying of the cellulose fibres. The number and size of the pores increases dramatically as well. Based on SEM images, we assumed the increase of porosity after PM for BC-AD would be greater because of the “brush” structure, which was confirmed by the BJH analysis—the porosity increased almost 13 times for BC-AD, and 6 times for BC-L. Thus, after PM the specific surface area of the BC-L and BC-AD reached comparable values. On the basis of these results, we can conclude that PM in combination with the drying process leads to the increase of pores and surface area, which results in significant changes in surface morphology which can improve cell adhesion.

### 3.6. Contact Angle and Hydrophilicity

The new morphology and higher oxygen content after PM can lead to changes in hydrophilicity of the material surface that can be represented by the water contact angle [59].

The contact angle measurement results (Table 4) showed that BC-AD is more hydrophobic than BC-L. The contact angles for those samples are (63.91 ± 2.69)° and (34.74 ± 6.8)°, respectively. These differences can be attributed to the different specific surface area, which is smaller for BC-AD. For hydrophilic materials (contact angle less than 90°) the higher porosity leads to a lower contact angle. This is due to the water drop being absorbed into the pores of a hydrophilic material. After PM, we observed further decrease of the contact angle that was greater in the case of BC-AD; both samples showed similar values after PM. This is consistent with much higher porosity increase for plasma-modified BC-AD compared to BC-L (Table 3). Additionally, an increase of surface oxygen content after PM represented by the O/C in XPS measurements (Table 2) leads to a more hydrophilic material. For comparison, Kurniawan et al. [45] also observed decrease of contact angle after PM with N_2_ and O_2_ plasmas while for CF_4_ plasma there was an increase. However, Pertile et al. [44] noticed an increase of contact angle after N_2_ PM. It is useful to note that each research group used different plasma discharge parameters.

### 3.7. In Vitro Tests of Cell Cultivation on BC Samples

For in vitro tests, we chose the BC-AD and BC-L samples, unmodified and after 240 s PM, in order to evaluate the effect of air drying, lyophilization, and PM on the colonization of the samples with human HaCaT keratinocytes.

**Figure 7:**
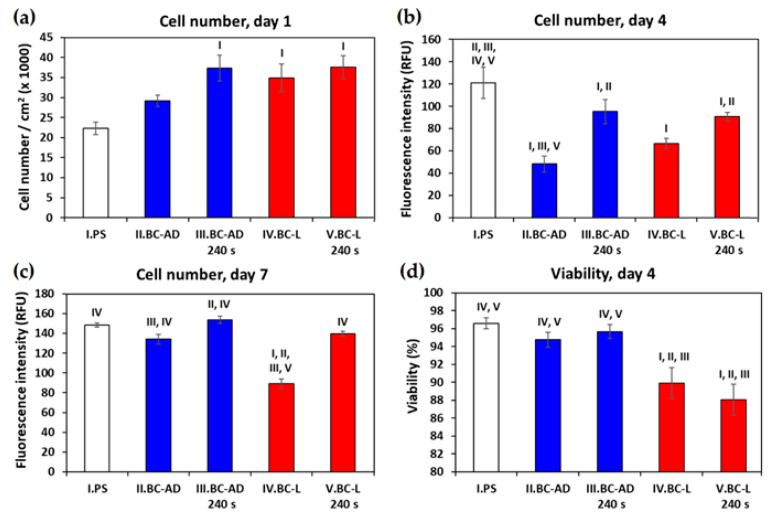
Number (**a**–**c**) and viability (**d**) of human HaCaT keratinocytes on days 1, 4 and 7 after seeding on control polystyrene wells (PS) or on BC. The data were obtained by (**a**) counting cells on 18–23 microscopic images, (**b**,**c**) measuring the intensity of fluorescence of cells stained with Hoechst 33258 on 7–20 microscopic images, or (**d**) trypan blue-exclusion test from four parallel samples of each experimental group. Mean ± S.E.M. (Standard Error of Mean), One way ANOVA, Student-Newman-Keuls Method. Statistically significant differences among the experimental groups (*p* ≤ 0.05) are indicated above the columns by numbers of differing groups.

**Figure 8:**
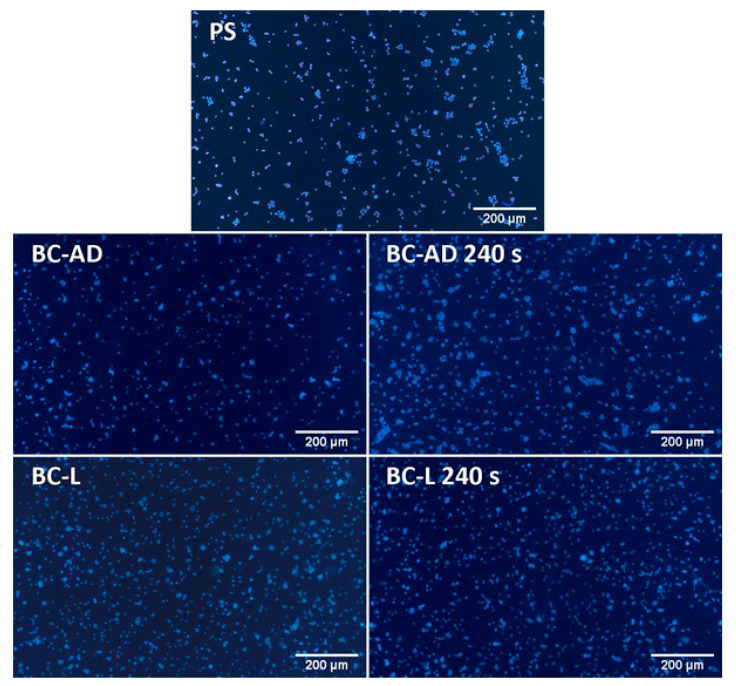
Human HaCaT keratinocytes on day 1 after seeding on control polystyrene wells (PS) or on BC. The cell nuclei were stained with Hoechst 33258. Olympus IX 51 epifluorescence microscope, DP 70 digital camera, obj. 4×, scale bar 200 µm.

On day 1 after seeding, the HaCaT cells on BC adhered generally in higher numbers than on the control PS wells, which was, however, more pronounced in BC-L than in BC-AD (Figure 7a). This result can be attributed to a higher porosity and a larger specific surface area of BC-L, which therefore provided more space for the initial cell attachment than BC-AD. Nevertheless, the initial adhesion of cells on BC-AD was significantly improved by PM, i.e., a technique which is generally used to enhance the attractiveness of various materials for cell adhesion. The main underlying mechanism of this improvement is increase in the material hydrophilicity, manifested by a significant decrease of water drop contact angle (from approx. 64° to 25°–33°; Table 4). On wettable materials, the cell adhesion-mediating proteins, such as fibronectin and vitronectin, spontaneously adsorb to the materials from biological fluids (including cell culture media), are attached in an active, physiological conformation, and are well-accessible for cell adhesion receptors (e.g., integrins) on cells [10,59,60]. BC-L was sufficiently wettable even before PM (contact angle of approx. 35°), and thus the PM did not further increase significantly the number of initially adhered cells, as observed on BC-AD samples. Moreover, the BC-AD samples modified with plasma were the first substrates on which the HaCaT cells started to form well-apparent and distinct islands typical for keratinocytes, which are important initial structures for creating a continuous cell layer (Figure 8).

**Figure 9:**
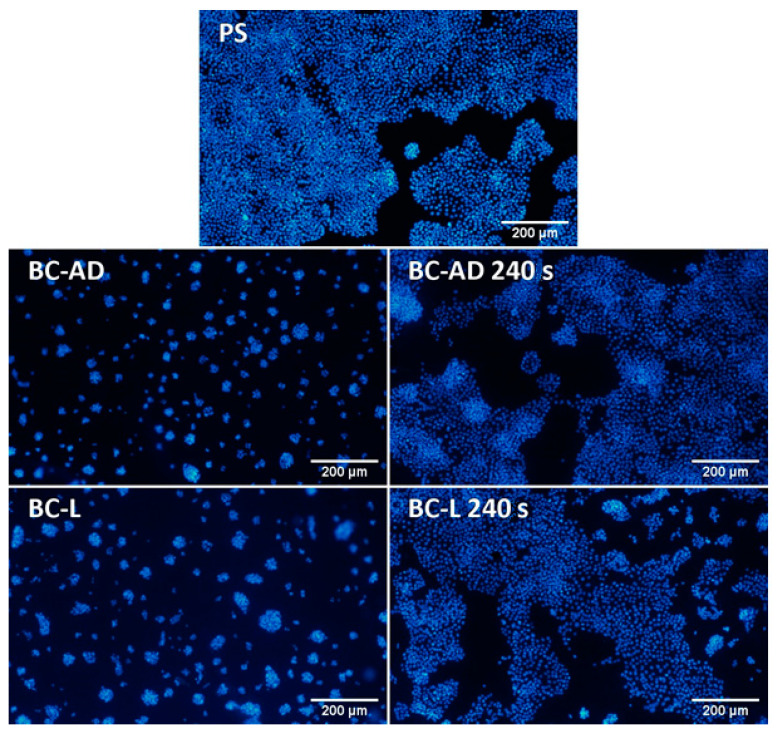
Human HaCaT keratinocytes on day 4 after seeding on control polystyrene wells (PS) or on BC. The cell nuclei were stained with Hoechst 33258. Olympus IX 51 epifluorescence microscope, DP 70 digital camera, obj. 4×, scale bar 200 µm.

On day 4 after seeding, however, the cell number became significantly lower on all tested BC samples in comparison with the reference PS wells (Figure 7b). In accordance with this, the cells on the images taken on day 4 covered a considerable part of the PS surface and were almost confluent, while the cells on the BC-AD and BC-L samples without PM were in an early phase of islet formation (Figure 9). This result can be explained by the fact that the flat PS surface provided a better support for the cell spreading (which is a prerequisite of the subsequent cell proliferation) than the rougher and more irregular BC surfaces. It was particularly apparent on BC-AD samples, which showed more rugged surface on SEM images (Figure 5). It is known from studies on osteoblasts that the increased material surface roughness often hampered proliferation of these cells [61,62]. This phenomenon could be even more pronounced in keratinocytes, which are epithelial cells with polarization (i.e., functional specialization) of their basal and apical cytoplasmic membrane, designated to cover surfaces of various organs, i.e., to live and grow in a 2D-like environment. In our earlier study, the negative effect of increased surface roughness on the cell proliferation was more apparent in endothelial cells, i.e., another type of epithelial-like cells, than in osteoblasts [63].

Nevertheless, the growth of HaCaT cells on BC-AD and BC-L was markedly improved by PM. On plasma-modified BC samples, the cells reached significantly higher cell numbers than on the unmodified samples (Figure 7b), and the cell islands on these samples developed into large cell colonies (Figure 9).

As revealed by a trypan blue exclusion test performed on day 4, the cells on the tested samples generally showed a high viability, ranging from approx. 88% to 97% (Figure 7d). Surprisingly, the lowest viability values were observed in cells cultivated on lyophilized BC, especially on samples modified with plasma. These samples contained the highest amount of oxygen (Table 2), which might be associated with damage to cells by reactive oxygen species. In addition, the surfaces of lyophilized samples are highly porous and contain numerous surface irregularities and areas of variable overall thickness and hardness, which can hamper cell spreading. It has been previously reported that keratinocytes prefer softer materials over harder ones [64,65]. Similarly, as mentioned above, the more rugged surface, which can be tolerated or even preferred by osteoblasts [66], can decrease the adhesion and proliferation of keratinocytes as well as other cells of soft tissues.

On day 7 after seeding, the cell number on the tested BC samples equalized with that on reference PS; i.e., the initial increase in cell number on BC samples, observed on day 1, was lost (Figure 7c). On BC-L samples, the final cell number was even significantly lower than on the other samples. This can be a consequence of the decreased cell viability observed on day 4 (Figure 7d), due to some negative effects of BC-L samples on cell spreading and proliferation, such as their surface irregularities and possible presence of oxygen radicals. Additionally, higher porosity (Table 3) of BC-L materials can lead to higher swelling of these materials in water-containing environments, such as cell culture media, which can have rather negative effect on the cell adhesion, because it can decrease the toughness (rigidity) of the substrate material. It is known that very soft and deformable materials, such as hydrogels, cannot resist the traction forces generated by cells during their spreading and support the viable growth of cells [67,68].

**Figure 10:**
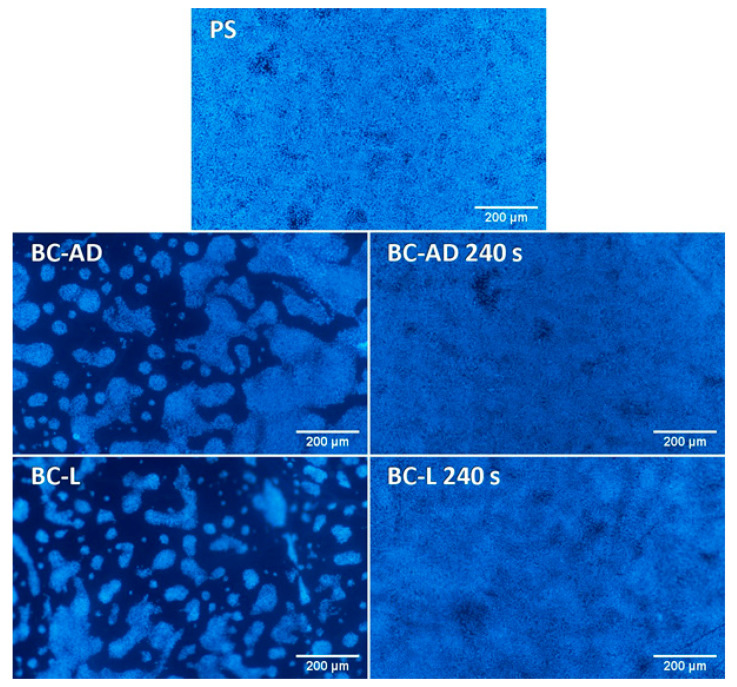
Human HaCaT keratinocytes on day 7 after seeding on control polystyrene wells (PS) or on BC. The cell nuclei were stained with Hoechst 33258. Olympus IX 51 epifluorescence microscope, DP 70 digital camera, obj. 4×, scale bar 200 µm.

Nevertheless, the cell colonization of all BC samples on day 7 significantly improved in comparison with the results from the 4th day of cultivation. From the images of cells on day 7 (Figure 10) it is evident that on PS and the plasma-modified BC, the cells are fully confluent, including those on BC-L, and in some parts of these samples, they created multilayer structures. On unmodified BC samples, the cells were still growing in colonies without reaching confluence, but these colonies have markedly enlarged in comparison with day 4.

Taken together, BC samples investigated in this study provided a good support for the adhesion, growth and viability of human HaCaT keratinocytes, comparable with standard tissue culture polystyrene, which is considered to be one of the most suitable materials for cell colonization. The effect of air drying and lyophilization on the cell colonization of BC was in general also comparable; it can be only distinguished that lyophilization had a positive effect on initial cell attachment, while the subsequent growth of cells was better on air-dried BC. However, the highest positive effect on the colonization of BC with cells has been provided by PM, as evident from the fact that the plasma-modified BC samples, either air-dried or lyophilized, were the only BC samples on which the cells were able to develop a continuous, confluent layer (an even multilayer) after one week of cultivation.

## 4. Conclusions

In our work, we described the differences between air drying and lyophilization with subsequent plasmatic modification of bacterial nanocellulose produced by the *Komagataeibacter sucrofermentans* bacteria strain (ATCC 700178). We found that BC-L materials maintained their structure, leading to higher porosity and specific surface area. Since the structure of BC-AD collapsed, the SEM revealed these samples to have a more rugged surface leading to almost two times higher contact angle. After plasmatic modification, the prepared samples showed a decrease of contact angle and increase of porosity and specific surface area. The O/C atomic ratio of the samples increased after PM, which suggests binding of the atmospheric oxygen to the activated surface immediately after modification. Compared to the literature, we obtained different values and trends of contact angles, morphology changes, and surface elemental composition. This is likely caused by the different parameters of the employed plasma discharge. Furthemore, we studied keratinocytes adhesion on our samples. We tested the initial adhesion (day 1) and subsequent growth (up to day 7) of human HaCaT keratinocytes on unmodified and plasma-modified BC-AD and BC-L. Due to its increased porosity and specific surface area, BC-L increased the initial adhesion of keratinocytes, but the subsequent cell growth was better on BC-AD. Modification with plasma for 240 s markedly accelerated the formation of typical keratinocyte islands and of continuous cell layers on both BC-AD and BC-L, which were comparable to those on standard cell culture polystyrene. Thus, the plasma-modified BC holds a great promise for skin tissue engineering and wound healing.

## Figures and Tables

**Table 1:** Gravimetric analysis of the BC weight loss after PM for different exposure times.

Sample	Weight Loss [%]
BC-AD 60 s	3.20 ± 0.36
BC-AD 240 s	14.26 ± 0.61
BC-AD 480 s	27.89 ± 2.88
BC-L 60 s	2.40 ± 0.45
BC-L 240 s	6.80 ± 1.27
BC-L 480 s	12.64 ± 3.00

**Table 2:** Carbon and oxygen atomic concentrations of BC samples measured by XPS with 0° detection angle.

Sample	C (%)	O (%)	O/C
BC-AD	55.80	44.20	0.6124
BC-AD 60 s	58.39	41.61	0.7126
BC-AD 240 s	59.49	40.51	0.6809
BC-L	57.24	42.76	0.6595
BC-L 60 s	59.36	40.64	0.6847
BC-L 240 s	49.69	50.31	1.0125

**Table 3:** Specific surface area (S_BET_) and total pore volume (V_p_) of BC samples measured by BET analysis.

Sample	S_BET_ [m^2^·g^−1^]	V_p_ [cm^3^·g^−1^]
BC-AD	9.9 ± 1.6	0.011 ± 0.002
BC-AD 240 s	140.5 ± 4.8	0.142 ± 0.008
BC-L	45.0 ± 0.7	0.056 ± 0.001
BC-L 240 s	156.3 ± 1.5	0.308 ± 0.015

**Table 4:** Values of water contact angle of BC samples measured by goniometry.

Sample	Contact Angle [°]
BC-AD	63.91 ± 2.69
BC-AD 60 s	25.42 ± 2.86
BC-AD 240 s	32.79 ± 2.00
BC-L	34.74 ± 6.80
BC-L 60 s	27.00 ± 2.80
BC-L 240 s	20.90 ± 1.90
